# Rescue of nucleus pulposus cells from an oxidative stress microenvironment via glutathione-derived carbon dots to alleviate intervertebral disc degeneration

**DOI:** 10.1186/s12951-024-02683-2

**Published:** 2024-07-12

**Authors:** Wenzhen Bu, Yu Shi, Xueping Huang, Shang Wu, Letao Jiang, Chun Pan, Dandan Li, Zhuobin Xu, Huihui Wang, Hao Chen, Jianwei Du

**Affiliations:** 1 Affiliated Hospital of Yangzhou University, Yangzhou University, Yangzhou, 225001 China; 2 Institute of Translational Medicine, Medical College, Yangzhou University, Yangzhou, 225001 China; 3 Jiangsu Key Laboratory of Integrated Traditional Chinese and Western Medicine for Prevention and Treatment of Senile Diseases, Yangzhou University, Yangzhou, 225001 China

**Keywords:** Glutathione-doped carbon dot nanozymes, Intervertebral disc degeneration, Reactive oxygen species, Oxidative stress, Cellular senescence, Inflammation

## Abstract

The senescence of nucleus pulposus (NP) cells (NPCs), which is induced by the anomalous accumulation of reactive oxygen species (ROS), is a major cause of intervertebral disc degeneration (IVDD). In this research, glutathione-doped carbon dots (GSH-CDs), which are novel carbon dot antioxidant nanozymes, were successfully constructed to remove large amounts of ROS for the maintenance of NP tissue at the physical redox level. After significantly scavenging endogenous ROS via exerting antioxidant activities, such as superoxide dismutase (SOD), catalase (CAT), glutathione peroxidase (GPx), and total antioxidant capacity, GSH-CDs with good biocompatibility have been demonstrated to effectively improve mitochondrial dysfunction and rescue NPCs from senescence, catabolism, and inflammatory factors in vivo and in vitro. In vivo imaging data and histomorphological indicators, such as the disc height index (DHI) and Pfirrmann grade, demonstrated prominent improvements in the progression of IVDD after the topical application of GSH-CDs. In summary, this study investigated the GSH-CDs nanozyme, which possesses excellent potential to inhibit the senescence of NPCs with mitochondrial lesions induced by the excessive accumulation of ROS and improve the progression of IVDD, providing potential therapeutic options for clinical treatment.

**Graphical Abstract:**

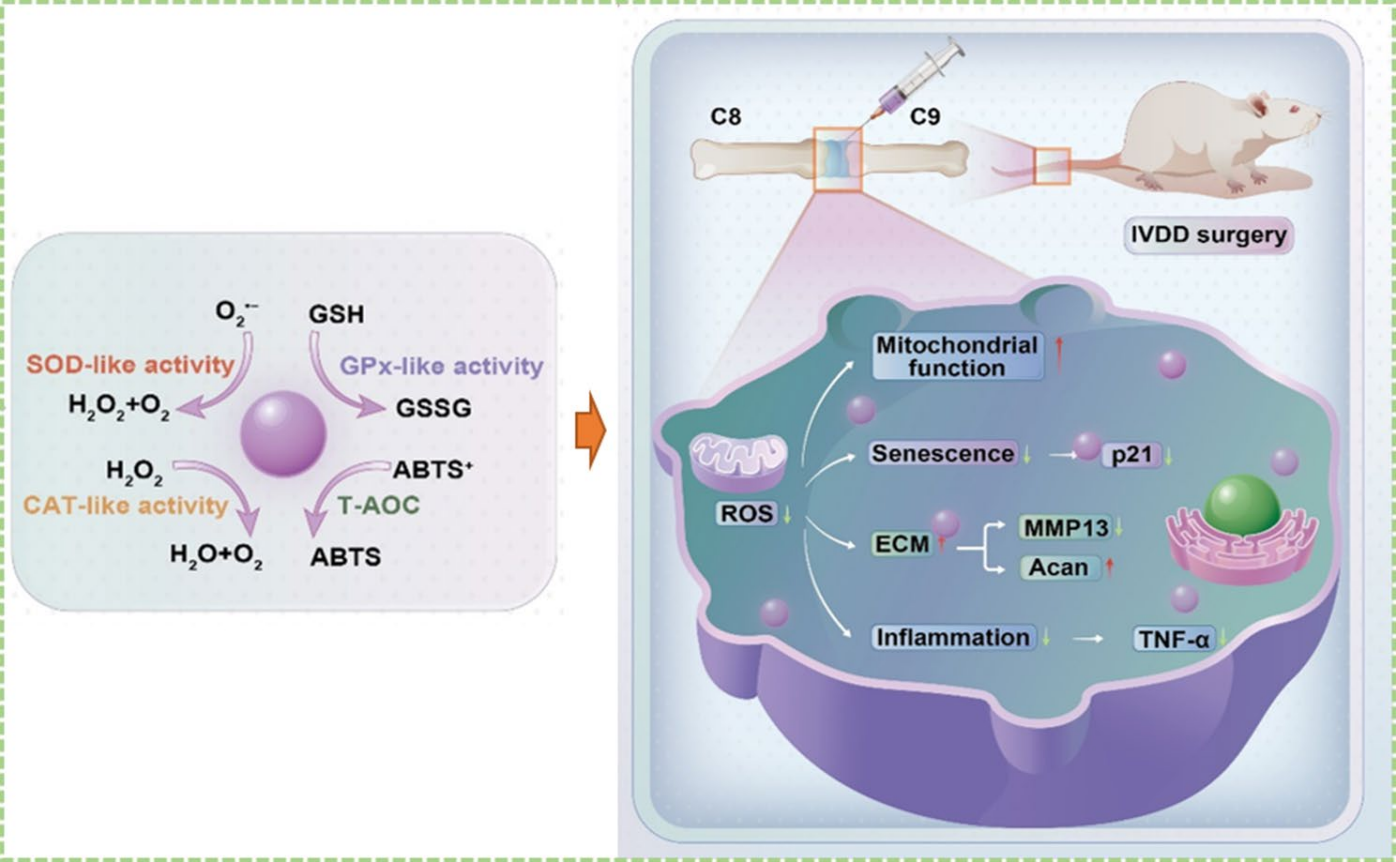

## Introduction

Presently, low back pain (LBP) has become a worldwide health problem that seriously impairs life quality and even increases the burden of the social economy and medical care [1]. What’s more, the phenomenon tends to occur in the younger generation. Conservative treatments such as the administration of painkillers and surgical treatments show confined therapeutic potential for further worsening of intervertebral disc degeneration (IVDD) [2, 3]. The intervertebral disc (IVD) is composed of the inner nucleus pulposus (NP), outer annulus fibrous (AF) and upper and lower cartilaginous endplates (CEPs). The inner NP is a gel-like tissue that is rich in randomly arranged collagen, such as aggrecan (Acan), and radially arranged elastin fibers, which provide a stable structural basis for bearing the spinal stress load and protecting nucleus pulposus cells (NPCs) [4–6]. Reactive oxygen species (ROS) are a class of highly active molecules containing oxygen, including singlet oxygen (^1^O_2_), hydrogen peroxide (H_2_O_2_), superoxide anion (O_2_^•−^) and hydroxyl radical (•OH) [7]. Studies have shown that the anomalous accumulation of ROS is an important detonator of NPCs senescence, subsequently leading to the incidence and progression of IVDD [8]. In addition, the pathological process of redox imbalance is also accompanied by enhanced catabolism, inflammation, and weakened metabolism, which will continually aggravate the senescence of NPCs [9–11]. NPCs senescence may gradually accelerate the loss of Acan and type II collagen (Col-2), which weakens the tolerance of NP tissues to mechanical loading, causing disorders such as spinal instability and neurogenic pain [12]. Consequently, effective clearance of ROS and improvement of the intracellular oxidative stress microenvironment in NPCs are essential for alleviating IVDD.

Natural antioxidant enzymes, such as superoxide dismutase (SOD), catalase (CAT) and glutathione peroxidase (GPx), exist widely in biological systems, but their applications are limited due to their high purification cost, harsh reaction conditions and instability. Nanozymes possess the advantages of high catalytic activity, stable physiological characteristics and easy large-scale production ability. As a new type of nanozyme, carbon dots (CDs) are widely used in nanomedicine because of their ultrasmall size, high stabilization, good biocompatibility and easy modification. Especially in the field of IVDD therapy, the ultra-small particle size facilitates its effective function in the harsh environment of high pressure, hypertonicity, and extreme lack of oxygen. Researches have demonstrated that CDs can effectively rescue degenerative discs via scavenging ROS [13]. However, the harsh therapeutic environment poses a huge obstacle for its adequate therapeutic effect, and the development of high-active CDs still remains a challenge. Glutathione (GSH) is a combination of glutamic acid, cysteine and glycine, in which the thiol group of cysteine is the active group of glutathione and plays an important role in the process of antioxidant defense [14]. Currently, researches have been performed to apply GSH into CDs to enhance the antioxidant-like activities. Tan et al. demonstrated that GSH CDs effectively alleviated cisplatin-induced ototoxicity via scavenging intracellular ROS [15]. However, excellent SOD activity alone is not sufficient to scavenge excessive ROS, and carbon-dot nanozymes with multiple cascade-like activities are of greater value for further research and development.

**Scheme 1:**
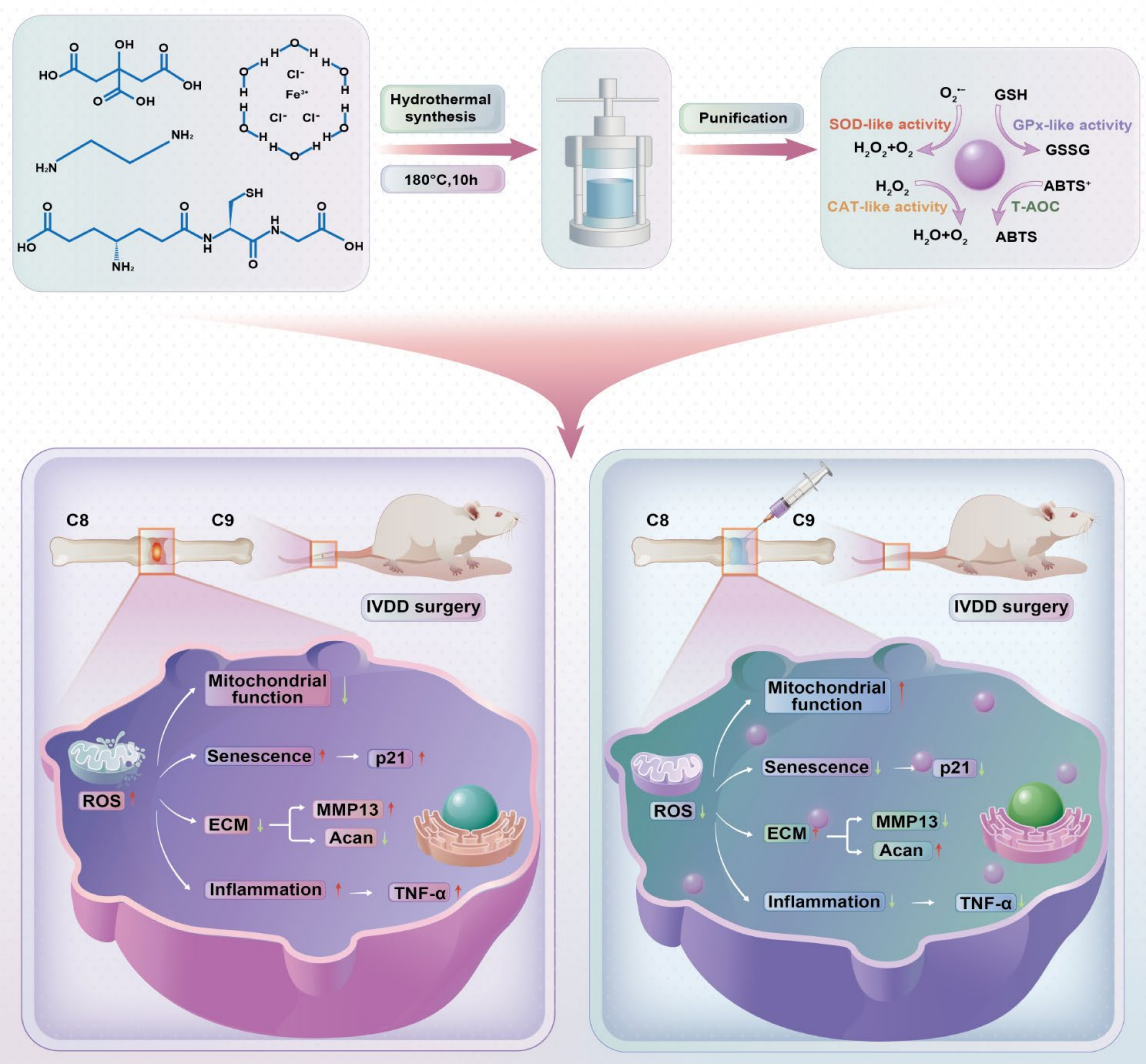
Schematic overview of the mechanism by which GSH-CDs reverse IVDD by balancing ROS and alleviating inflammation and senescence in NPCs

In this research, a novel carbon dot nanozyme, namely, GSH-derived carbon dots (GSH-CDs), was prepared for the harsh oxidative stress microenvironment in IVDD. GSH-CDs had been demonstrated to exhibit excellent SOD, CAT and GPx-like enzyme activities, and were applied in vitro and in vivo models to exert antioxidant effects by inhibiting oxidative stress impairment and NPCs senescence induced by ROS to delay IVDD (Scheme 1). Our study provides valuable insights for the development of novel CDs with highly efficient antioxidant enzyme activity, providing new ideas for the application of CDs in the treatment of IVDD.

## Results and discussion

### Characterization of the GSH-CDs

**Fig. 1:**
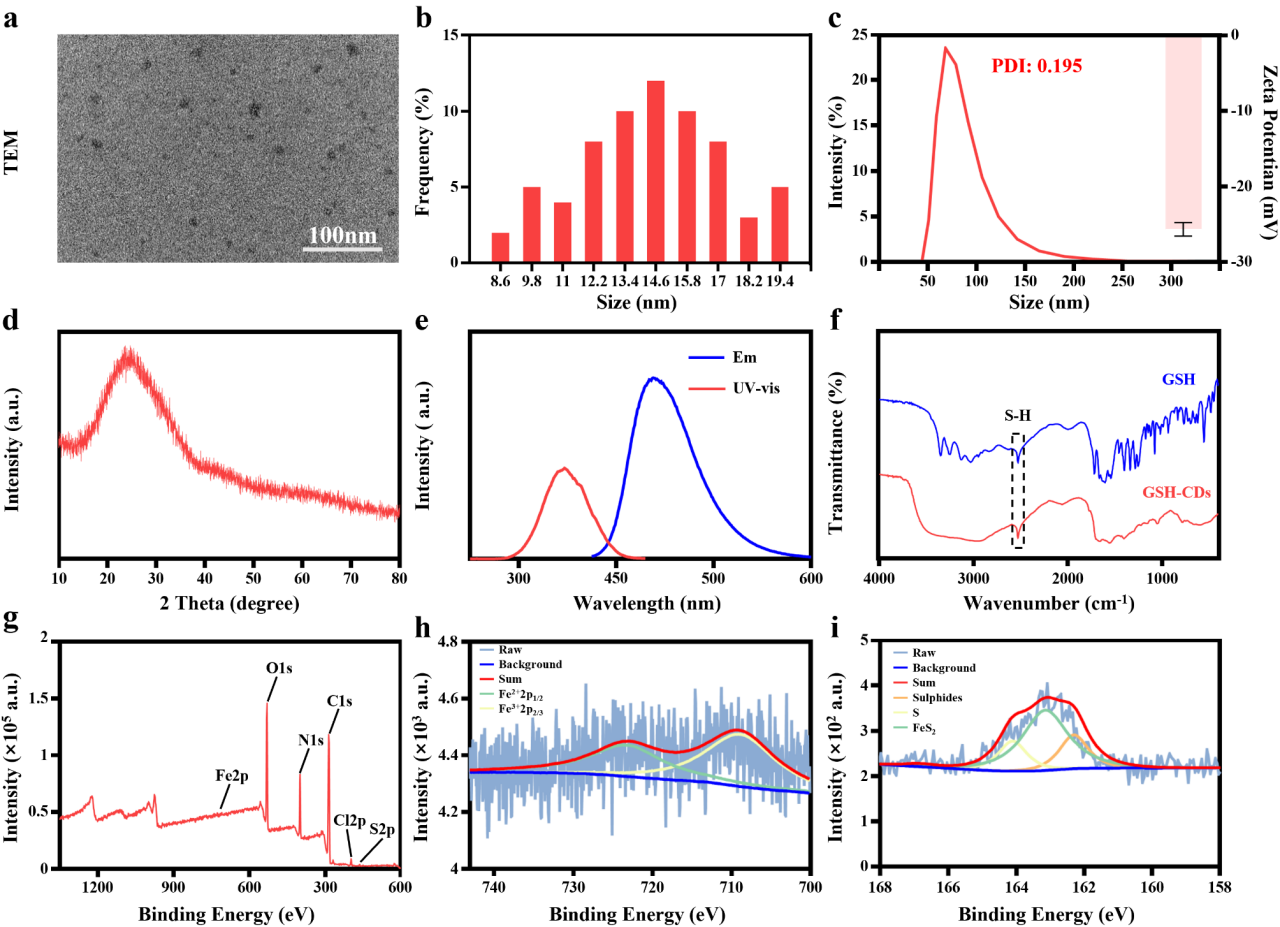
Characterization of GSH-CDs. (**a**) TEM image and (**b**) size distribution of the GSH-CDs (Scale bar = 100 nm). (**c**) Hydrated particle size (left) and zeta potential (right) of GSH-CDs. (**d**) XRD pattern of GSH-CDs. (**e**) UV‒visible absorption and fluorescence emission spectra of GSH-CDs. (**f**) FT-IR spectra of GSH and GSH-CDs. (**g**) XPS spectrum of GSH-CDs. (**h**) XPS Fe 2p spectra of GSH-CDs. (**i**) XPS S 2p spectra of GSH-CDs

In this study, GSH-CDs were prepared via a hydrothermal synthesis method. Transmission electron microscopy (TEM) images revealed the uniformly distributed orbicular-like morphology of GSH-CDs, with a particle size of approximately 14 nm (Fig. 1a, b). The average hydrated size of GSH-CDs was 82.65 nm (Fig. 1c, left), with a zeta potential of -35.67 ± 0.88 mV (Fig. 1c, right), indicating prominent dispersity in aqueous media. The XRD pattern demonstrated that GSH-CDs exhibited no characteristic peaks, indicating low crystallinity (Fig. 1d). To understand the optical properties of GSH-CDs, UV‒visible (UV‒Vis) and fluorescence emission spectra were measured. The spectrum of GSH-CDs showed a typical absorption peak near 347 nm and a maximum emission wavelength of 440 nm (Fig. 1e). Fourier transform infrared spectroscopy (FT-IR) of GSH-CDs and GSH was performed to determine the functional groups on the surface of GSH-CDs. As shown in Fig. 1f, the absorption band at approximately 2653 cm^− 1^ corresponding.

to the stretching vibrations of S-H showed that the surface of GSH-CDs contained sulfhydryl groups, indicating that GSH-CDs partially retained the structural properties of GSH. In addition, the chemical structure and composition of the GSH-CDs were investigated using X-ray photoelectron spectroscopy (XPS), which revealed the presence of C (62.0%), N (15.8%), O (18.9%), Fe (0.2%), and S (2.1%) elements in the GSH-CDs (Fig. 1g). The Fe 2p high-resolution spectra of GSH-CDs (Fig. 1h) indicated the presence of Fe^3+^ 2p_3/2_ (710.9 eV) and Fe^2+^ 2p_1/2_ (723.4 eV). The S2p high-resolution spectra (Fig. 1i) indicate the presence of S (164.1 eV), FeS_2_ (163.2 eV), and sulfides (162.3 eV). The above results suggest the successful construction of GSH-CDs with good water solubility and good optical properties.

### Multiple enzyme-like activities of GSH-CDs

**Fig. 2:**
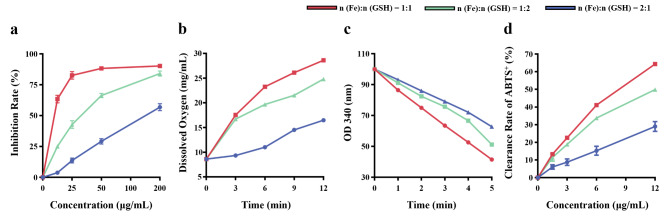
Enzyme-like activity of GSH-CDs. (**a**) SOD-like enzyme activity of the GSH-CDs. (**b**) CAT-like enzyme activity of the GSH-CDs. (**c**) GPx-like enzyme activity of the GSH-CDs. (**d**) Determination of the total antioxidant enzyme activity of the GSH-CDs

When environmental stress occurs, such as nutritional starvation and hypoxia, homeostasis in NPCs is out of balance, reflected by insufficient cellular antioxidant capacity and excessive ROS production [16]. Therefore, keeping the dynamic balance of ROS plays an important role in maintaining NPCs stability. SOD and CAT, which can effectively remove ROS, such as ·O_2_^−^ and H_2_O_2_, are considered typical antioxidant enzymes with specific physiological activity [17–20]. In particular, SOD-like enzyme activity is crucial for the biomedical application of antioxidant nanomaterials, which has been successfully used in disease models such as acute kidney injury septicemia, stroke, et al [21–23]. In this regard, the superoxide anion scavenging capacity of GSH-CDs was assayed utilizing a SOD enzyme activity kit. It showed a concentration correlation with the percentage inhibition of O_2_^•−^. In addition, GSH-CDs synthesized with a 1:1 ratio of FeCl_3_·6H_2_O and GSH showed the best SOD-like activity with 260.1 U/mg, which was superior to that of 1:2 ratio (78.1 U/mg) and 2:1 ratio (32.5 U/mg) (Fig. 2a). CAT catalyzes the decomposition of H_2_O_2_ to O_2_ and H_2_O, protecting organisms from H_2_O_2_-induced oxidative stress damage. According to the data recorded by the dissolved oxygen meter, the amount of O_2_ produced by GSH-CDs gradually increased over time. Moreover, GSH-CDs synthesized with a 1:1 ratio exhibited superior O_2_ catalytic efficiency (approximately 28.24 mg/L) at 37 °C for 12 min (Fig. 2b). Moreover, when reacting with free radicals, intracellular lipids can cause oxidative damage and even cell death owing to the production of lipid peroxides. GPx plays an important role in maintaining ROS metabolic homeostasis by eliminating free radicals. As shown in Fig. 2c, GSH-CDs also exhibited prominent GPx-like activity, reducing lipid peroxides effectively in a dose- and time-dependent manner. Likewise, the GSH-CDs synthesized with a 1:1 ratio possessed the best GP_X_-like activity according to the faster decrease in OD 340. In addition, the total antioxidant capacity of GSH-CDs increased significantly with increasing concentration, and the GSH-CDs synthesized at a ratio of 1:1 showing the excellent capacity (Fig. 2d). Hence, GSH-CDs synthesized at a ratio of 1:1 exhibited outstanding antioxidant enzyme activities, providing a favorable basis for alleviating IVDD caused by oxidative stress.

### GSH-CDs can rescue the degeneration of NPCs

**Fig. 3:**
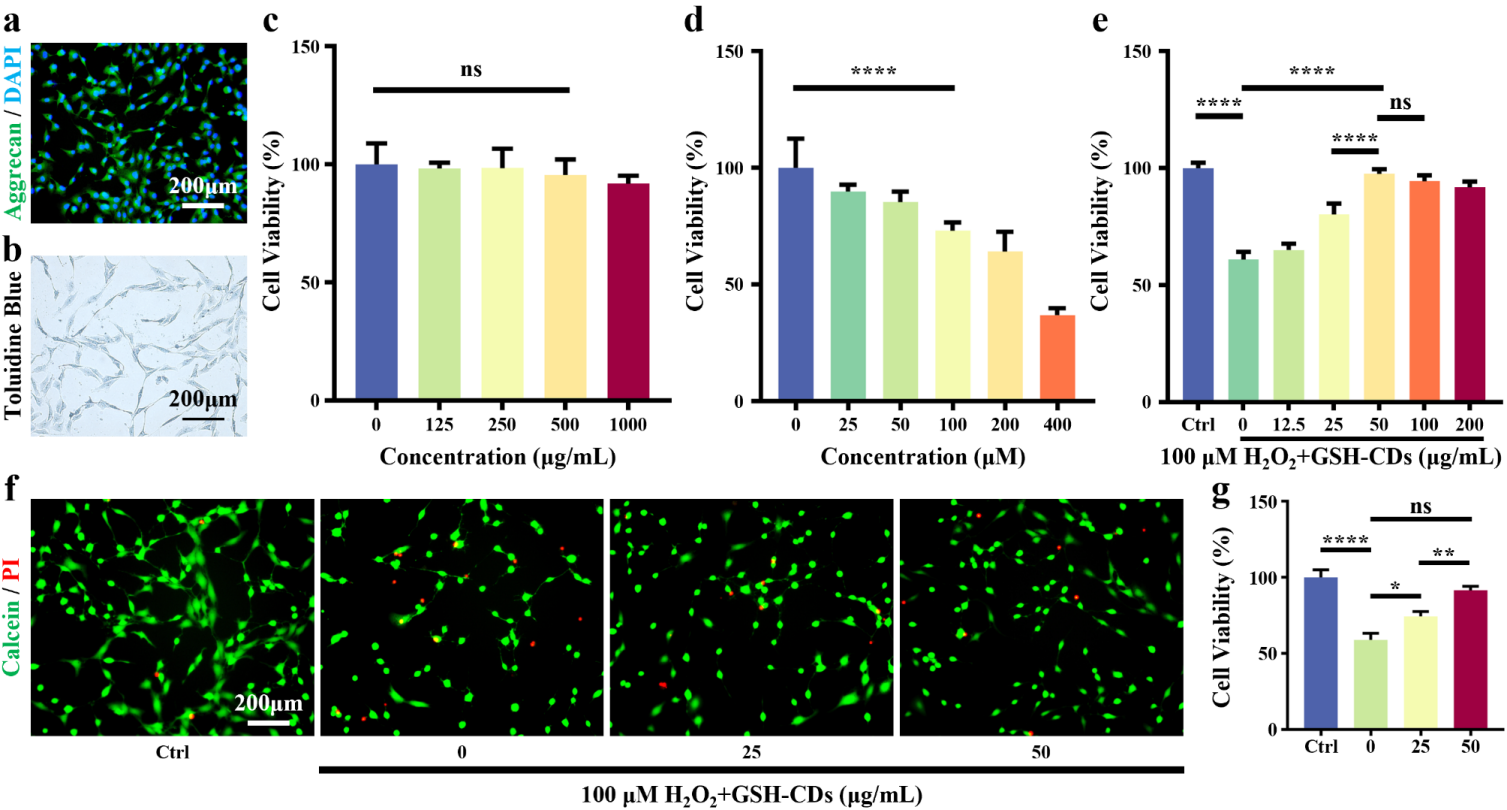
Biocompatibility and rescue ability of GSH-CDs. (**a**) Immunofluorescence staining of Aggrecan. (Scale bar = 200 μm). (**b**) Toluidine blue staining (Scale bar = 200 μm). (**c**) Cell viability of NPCs incubated with different concentrations of GSH-CDs. (**d**) Cell viability of NPCs incubated with different concentrations of H_2_O_2_. (**e**) The protective effect of GSH-CDs against H_2_O_2_-induced oxidative stress in NPCs. Calcein/PI staining (**f**) of NPCs cocultured with GSH-CDs and H_2_O_2_ and semiquantitative analysis (**g**) of the different groups. (* indicates *p* value < 0.05, ** indicates *p* value < 0.01, **** indicates *p* value < 0.0001)

For in vitro experiments, NPCs were extracted from rat tail vertebrae and successfully identified via toluidine blue (TB) staining and aggrecan immunofluorescence staining (Fig. 3a, b). To detect the cytotoxicity of GSH-CDs, the viability of NPCs was tested via CCK8 assay. As shown in Fig. 3c, GSH-CDs had little toxicity with a very high safe concentration (approximately 500 µg mL^− 1^). Hydrogen peroxide has been shown to be a commonly used means of modelling the microenvironment of oxidative stress in NPCs [24, 25]. Firstly, NPCs were cocultured with H_2_O_2_ at various concentrations. As presented in Figs. 3d and 100 µM H_2_O_2_, which was selected as the induction concentration for the subsequent in vitro experiments. To further explore the rescue effect of GSH-CDs on H_2_O_2_-induced oxidative stress impairment in NPCs, the cells pretreated with different concentrations of GSH-CDs were then cocultured with H_2_O_2_ (100 µM) for 24 h. GSH-CDs (50 µg mL^− 1^) had the greatest inhibitory effect on H_2_O_2_-induced oxidative stress in NPCs (Fig. 3e). Similarly, the results of calcein/PI staining and semiquantitative fluorescence analysis showed that GSH-CDs significantly rescued NPCs from death in an H_2_O_2_-induced oxidative stress microenvironment (Fig. 3f, g). In brief, GSH-CDs, which have high biocompatibility, can protect NPCs from H_2_O_2_-induced oxidative stress in vitro by exerting extraordinary antioxidant enzyme-like activity.

### Effect of GSH-CDs on mitochondrial dysfunction and cellular senescence

**Fig. 4:**
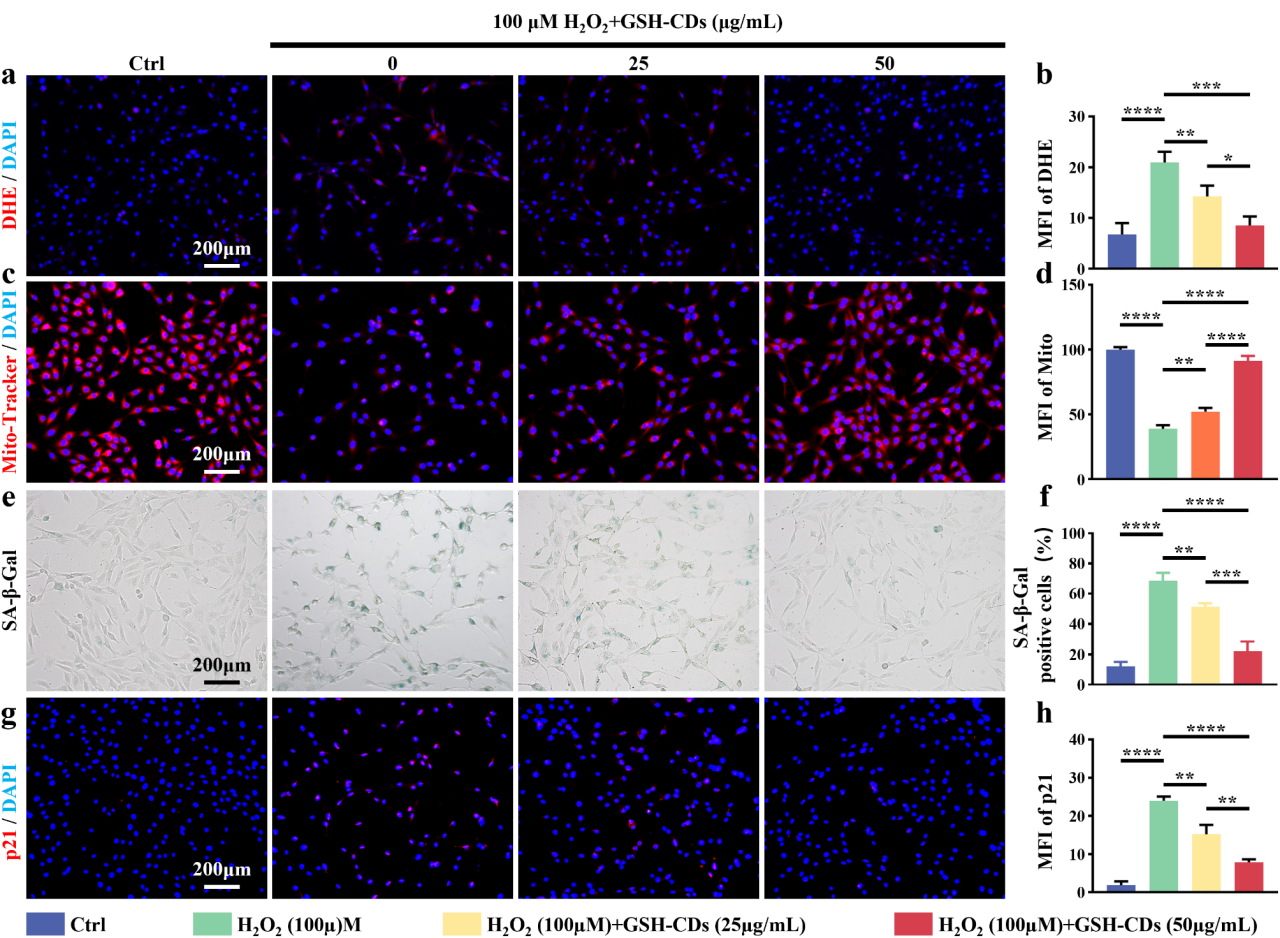
Rescue effect of GSH-CDs on NPCs senescence and mitochondrial dysfunction via the removal of excessive ROS. (**a**) DHE fluorescence staining images of NPCs in different groups (Scale bar = 200 μm). (**b**) Semiquantitative analysis of DHE staining. (**c**) Images of mitochondrial fluorescence staining (Scale bar = 200 μm). (**d**) Semiquantitative analysis of mitochondrial fluorescence intensity. SA-β-gal staining (**e**) of NPCs and semiquantitative analysis (**f**) of the percentage of SA-β-Gal-positive cells in different groups (Scale bar = 200 μm). (**g**) Immunofluorescence staining results of p21 in different groups (Scale bar = 200 μm). (**h**) Semiquantitative analysis of the fluorescence intensity of p21. (* indicates a *p* value < 0.05, ** indicates a *p* value < 0.01, *** indicates a *p* value < 0.001, **** indicates a *p* value < 0.0001)

DHE fluorescent probe was used to demonstrate the protective effect of GSH-CDs against ROS. As shown in Fig. 4a and **b**, the fluorescence intensity of H_2_O_2_-treated NPCs was significantly higher than that of control group, indicating that H_2_O_2_ significantly increased ROS levels in H_2_O_2_-treated NPCs. Moreover, the red fluorescence intensity significantly decreased in the GSH-CD-pretreated groups, indicating GSH-CDs effectively inhibited ROS production. In addition, the high-concentration group (50 µg mL^− 1^) showed a more significant therapeutic effect than the low one. Changes in the mitochondrial membrane potential (MIMP) reflect mitochondrial functional status, which is inextricably associated with ROS-induced dysregulation of redox balance homeostasis. Therefore, a mitochondrial red fluorescent probe (CMXRos) was used to specifically label the bioactive mitochondria in NPCs and detect the MIMP level. CMXRos staining revealed that GSH-CDs significantly improved H_2_O_2_-mediated inhibition of MIMP in NPCs, and this effect was even more pronounced when the concentration of GSH-CDs increased (Fig. 4c, d). Disequilibrium in mitochondrial redox status is known to be significantly associated with the occurrence and development of cellular senescence [26, 27]. Therefore, SA-β-gal staining was performed to verify the suppressive effect of GSH-CDs on NPCs senescence. There was an increase in the number of SA-β-Gal-positive cells in the H_2_O_2_-induced oxidative stress microenvironment, while GSH-CDs efficiently suppressed this phenomenon (Fig. 4e, f). Moreover, for the senescence-specific marker p21, similar trends were observed in the immunofluorescence staining results (Fig. 4g, h). The above results suggested that GSH-CDs have the potential in alleviating H_2_O_2_-induced NPCs senescence.

### GSH-CDs ameliorate the anabolic and catabolic balance of NPCs in vitro

**Fig. 5:**
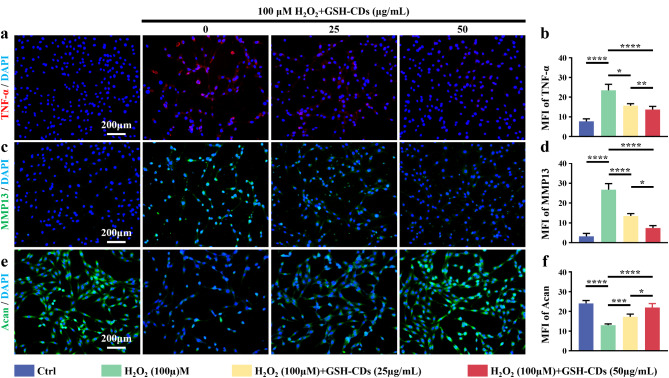
GSH-CDs re-establish anabolic and catabolic balance to maintain NPC homeostasis. **a**–**c**) Immunofluorescence staining results of TNF-𝛼, MMP13, and Acan in different groups (Scale bar = 200 μm). **d**–**f**) Semiquantitative analysis of the fluorescence intensity of TNF-𝛼, MMP13, and Acan. (* indicates a *p* value < 0.05, ** indicates a *p* value < 0.01, *** indicates a *p* value < 0.001, **** indicates a *p* value < 0.0001)

Cellular senescence induced by ROS initiation and development is always accompanied by the secretion of proinflammatory cytokines, extracellular matrix degradation proteins and chemokines, which are collectively referred to as senescence-associated secretory phenotypes (SASPs) [28, 29]. After H_2_O_2_ induction, the expression of proinflammatory cytokines (such as TNF-𝛼) and catabolism-related proteins (such as MMP13) was significantly increased in NPCs, accompanied by decreased anabolic-related protein (such as aggrecan) expression, revealing the disruption of homeostasis within NPCs (Fig. 5a-f). Satisfactorily, adequate homeostasis within NPCs was significantly rebalanced by GSH-CDs in a concentration-dependent manner. These results demonstrate the ability of GSH-CDs to ameliorate ROS-induced microenvironmental disorders in NPCs.

### GSH-CDs ameliorate the progression of IVDD in vivo

**Fig. 6:**
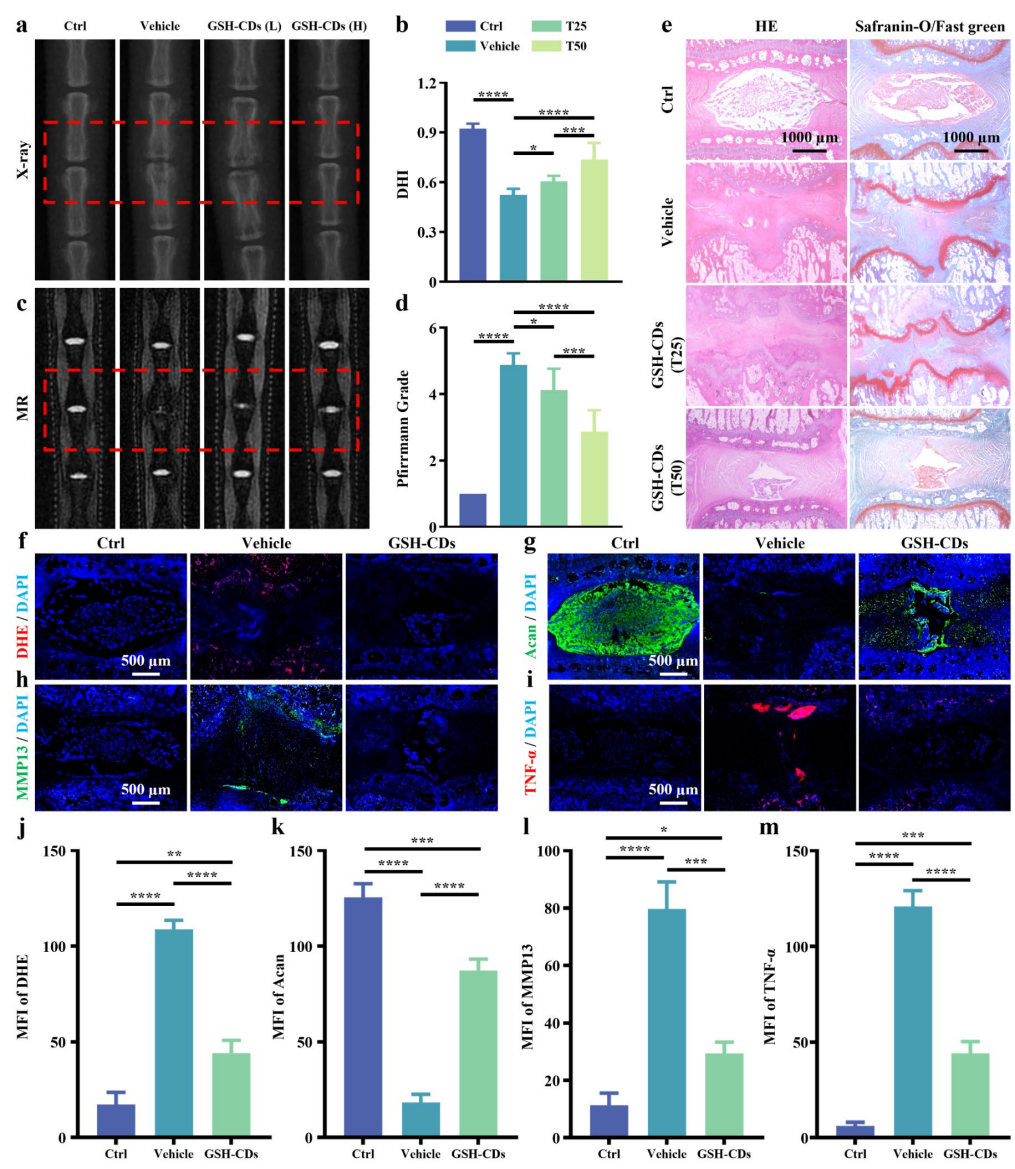
Imaging and histological changes in the caudal vertebrae of rats. (**a**) X-ray images and (**b**) DHI of the control group, vehicle group, L-GSH-CD group, and H-GSH-CD group. (**c**) MR images and (**d**) Pfirrmann grades of the different groups. (**e**) HE and SOFG staining of different groups (Scale bar = 1000 μm). Images of immunofluorescence fluorescence staining of DHE (**f**), Acan (**g**), MMP13 (**h**), and TNF-α (**i**) (scale bar = 500 μm) and semiquantitative analysis of the fluorescence intensity of DHE (**j**), Acan (k), MMP13 (**l**), and TNF-α (**m**) in the control group, vehicle group, and GSH-CD group. (* indicates a *p* value < 0.05, ** indicates a *p* value < 0.01, *** indicates a *p* value < 0.001, **** indicates a *p* value < 0.0001)

A needle puncture model was used to induce IVDD in rats to validate the in vivo therapeutic efficacy of GSH-CDs. After puncture, the uniformly dispersed GSH-CDs solution (0.714 µg kg^− 1^ or 1.43 µg kg^− 1^) was injected into the intervertebral disc, and PBS solution was injected into the control group. The height and composition of the intervertebral disc vary as IVDD progresses. Hence, imaging and histological assessments are reliable indicators of IVDD. X-ray and MRI results were obtained 4 weeks postoperatively. X-ray analysis revealed severe height loss and endplate boundary destruction in the puncture degeneration group, and these effects were alleviated to varying extents by local injection of different concentrations of GSH-CDs (Fig. 6a). In addition, according to the variation in the disc height index (DHI), an objective indicator reflecting the degree of IVDD, the GSH-CD group (1.43 µg kg^− 1^) exhibited greater improvement than the low group (0.714 µg kg^− 1^) (Fig. 6b). Furthermore, the Pfirrmann grading score calculated from the T_2_-weighted MR signal reliably reflects variations in the extent of IVDD [30]. Compared with that in the normal control group, water content in the vehicle group sharply decreased, while for the in vivo local treatment of the GSH-CDs, there was a greater salvage effect in the high GSH-CD group than the low group (Fig. 6c, d). According to the histomorphological evaluation of HE and SOFG staining, the IVD structure was severely damaged in the vehicle group, with significantly reduced or even no NP tissue. Fortunately, the GSH-CDs had a significant inhibitory effect on IVDD, which was reflected by the more intact cartilaginous structure and morphological characteristics in comparison to those of the vehicle group (Fig. 6e). In addition, to further verify the in vivo antioxidant and anti-inflammatory effects of GSH-CDs, immunofluorescence staining of several indicators of intervertebral disc tissue sections was performed. The tendency of the red fluorescence intensity of the DHE probe to indicate ROS impairment was again confirmed by the in vivo protective effect of GSH-CDs (Fig. 6f). Regarding the in vivo variation in the SASP, the expression of TNF-𝛼 and MMP13 was also significantly increased in the vehicle group, accompanied by decreased expression of aggrecan, demonstrating disruption of IVD homeostasis. Simultaneously, local application of GSH-CDs significantly reversed the endo-environmental imbalance (Fig. 6g-i). The results of the semiquantitative analysis of the fluorescence intensity of DHE, Acan, TNF-𝛼 and MMP13 are shown in Fig. 6j-m. Overall, GSH-CDs significantly rescued the development of IVDD in vivo through the removal of ROS and ameliorated the imbalance between anabolism and catabolism within the nucleus pulposus tissue.

### Metabolism and biocompatibility of GSH-CDs in vivo

In this study, GSH-CDs were shown to have therapeutic effects via local injection into the confined disc microenvironment. However, the in vivo metabolic behavior and biocompatibility of GSH-CDs still require further and longer-term research and verification. The pharmacokinetic.

**Fig. 7:**
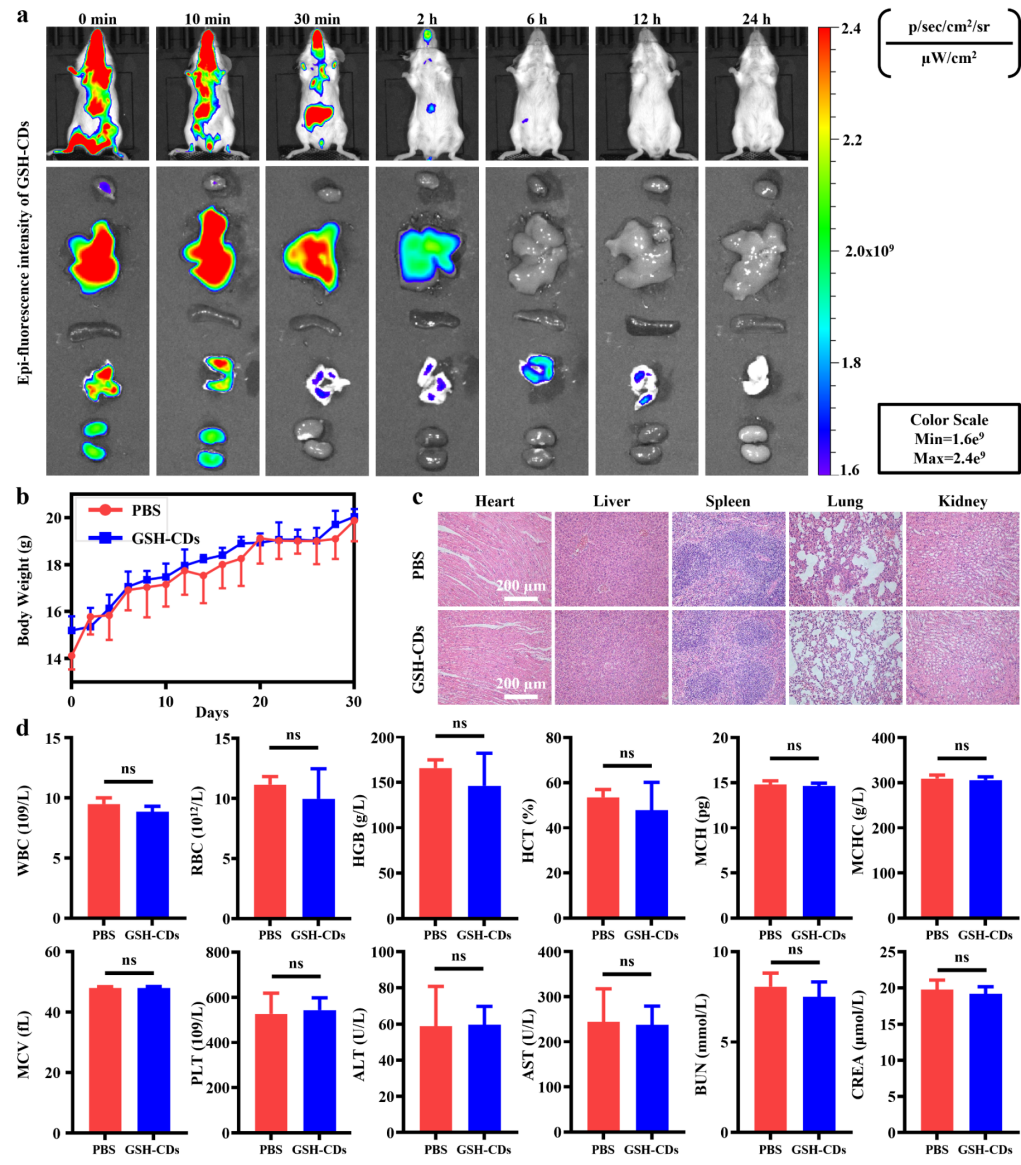
Biocompatibility and metabolic pathway of GSH-CDs in mice. (**a**) In vivo fluorescence images of GSH-CDs at different time intervals. (**b**) Trends of weight change within 30 days after tail vein injection of PBS or GSH-CDs. (**c**) HE staining of major organs (heart, liver, spleen, lungs and kidneys) in the PBS and GSH-CD groups after intravascular injection (Scale bar = 200 μm). (**d**) Hematological and biochemical indexes of each group

features of GSH-CDs modified with a red fluorescent indicator Ce6 were monitored *by* tracking the primary distribution and variation trend of the red fluorescence intensity in healthy BALB/c mice. After intravenous injection, GSH-CDs (5 mg kg^− 1^) were absorbed and then distributed to various organs via blood circulation. Initially, significant accumulation of GSH-CDs mainly occurred in the liver, lung, and kidney. Over time, the GSH-CDs were thoroughly metabolized within 24 h (Fig. 7a). To observe in vivo long-term biocompatibility, the body weights of the mice were measured every other day for one month after intravenous injection of GSH-CDs (5 mg kg^− 1^) or PBS. There was no significant difference in the weight variation between the two groups, indicating the prominent biocompatibility of GSH-CDs (Fig. 7b). In addition, tissue specimens of major organs and venous blood were collected 30 days after injection for HE staining and hematology examination, respectively. Similarly, there were no significant differences in histological morphology or hematological parameters between GSH-CDs and PBS (Fig. 7c, d). Taken together, these results indicated that the GSH-CDs possessed good biocompatibility with a rapid metabolic rate.

In this work, GSH-CDs was demonstrated as an effective candidate for delaying disc degeneration, which effectively improves the conditions of malignant oxidative stress within the NPCs microenvironment in a multiple cascade-like activities manner, that is, inhibits the senescence of NPCs induced by excessive accumulated ROS via exerting multiple antioxidant enzyme activity. It is hypothesized that this multiple antioxidant-like enzyme activity arises mainly from the incorporation of GSH. Firstly, as a natural tripeptide present in cells with excellent biocompatibility and bioactivity, GSH plays a central role in the cellular antioxidant defense system. Hence, GSH inherently possesses the ability to scavenge ROS. Secondly, by transforming it into nanomaterials, not only could its ROS-scavenging properties be retained, but this ability might also be enhanced due to the unique physicochemical properties of the nanomaterials. In addition, due to the advantage of small size (approximately 14 nm), GSH-CDs could be more readily absorbed and thus more efficiently function as ROS scavengers in the confined microenvironment of the intervertebral disc without exerting excessive pressure on the disc tissue. Satisfactorily, GSH-CDs inhibited NPCs from senescence via effectively scavenging ROS, thus significantly delaying the progression of IVDD. However, there still exists a few limitations in this research. Firstly, an SD rat model was employed in this research for IVDD induction and treatment. Although this animal model is valuable, they may not adequately mimic the complexities of disc degeneration in humans, such as age, gravitational forces, and strenuous activity. Besides, the duration of this research might be relatively short to capture long-term effects or the potential for GSH-CDs to be associated with delayed adverse effects. Thirdly, the exact mechanisms by which GSH-CDs exert their effects on IVDD might require more detailed investigation, including potential interactions with other cellular pathways. Finally, the long-term safety and potential side effects in practical applications need to be further investigated. Although GSH-CDs has been demonstrated with good performance in in vitro experiments, their pharmacokinetic properties, distribution, metabolism, and excretion in vivo also need further evaluation.

## Materials and methods

### Reagents and antibodies

Glutathione (GSH), citric acid, iron trichloride hexahydrate (FeCl_3_·6H_2_O), and Safranin O-Fast Green were acquired from Sigma‒Aldrich (China). Ethylenediamine (EDA) was obtained from Sinopharm (China). Sephadex G25 resin was purchased from Cytiva (China). Dialysis membranes (MWCO 500～1000) were purchased from Yuanye (China). A superoxide dismutase (SOD) kit was purchased from Cominbio (China). The Glutathione Peroxidase Assay Kit with NADPH, Total Antioxidant Capacity Assay Kit with a Rapid ABTS method, Cell Counting Kit-8, Calcein/PI Cell Viability/Cytotoxicity Assay Kit, MitoTracker Red CMXRos, Dihydroethidium (DHE), 4% Paraformaldehyde, DAPI dihydrochloride, Dimethyl sulfoxide (DMSO), and Hematoxylin and Eosin Staining Kit were purchased from Beyotime (China). Hydrogen peroxide (H_2_O_2_), pentobarbital sodium, and chlorin E6 (Ce6) were purchased from Aladdin (China). Aggrecan was purchased from ABclonal (China). Matrix metalloproteinase-13 (MMP13), goat anti-mouse IgG H&L, goat anti-rabbit IgG H&L, and TNF-α were purchased from Proteintech (China). p21^Waf1/Cip1^ was purchased from Santa Cruz Biotechnology (China). Dulbecco’s modified Eagle’s medium/nutrient mixture F12 (DMEM/F12) and fetal bovine serum (FBS) were purchased from Gibco (America). Phosphate-buffered saline (PBS), bovine serum albumin, antibody diluent, trypsin-EDTA solution, EDTA decalcifying solution, Senescence-Associated-β-Galactosidase (SA-β-Gal) Stain Kit, and toluidine blue (TB) cartilage stain solution were purchased from Solarbio (China).

### Instruments

The micromorphology of the GSH-CDs was evaluated via transmission electron microscopy (TEM, Tecnai 12, Philips). The zeta potentials and hydrated particle size of the GSH-CDs were detected by a Malvern ZEN3690 zeta sizer (Malvern, UK). The main functional groups and chemical bonds of the GSH-CDs were measured via a Cary 610/670 microinfrared spectrometer (Varian). The X-ray powder diffraction (XRD) pattern of the GSH-CDs was obtained using an X-ray diffractometer (D8 Advance, Bruker AXS). The UV–vis absorption and fluorescence emission spectra of the GSH-CDs were recorded on a multimode microplate reader (TECAN, Switzerland). X-ray photoelectron spectroscopy (XPS) of the GSH-CDs was performed using an X-ray photoelectron spectrometer (ESCALAB 250Xi, Thermo Scientific). Fluorescence images of mice injected with GSH-CDs were obtained with an IVIS Lumina Series III in vivo animal imaging system (Perkin-Elmer, USA).

### Synthesis of GSH-CDs

#### Preparation of GSH-CDs

GSH-CDs were prepared via a hydrothermal synthesis method. Briefly, CA, EDA, FeCl_3_·6H_2_O and GSH at different molar ratios (10:10:1:2, 10:10:2:2, and 10:10:2:1, respectively) were completely dissolved in 20 ml of ultrapure water under homogeneous stirring at room temperature for 30 min. The mixed solution was transferred to a reaction kettle at 180 °C for 10 h. After naturally cooling to room temperature, the resulting solution was filtered through a microporous filter (0.22 μm) to remove insoluble impurities. After dialysis purification, the final GSH-CD powder was collected via lyophilization.

#### Preparation of GSH-CDs-Ce6

Ce6 (20 mg, 0.0335 mmol, MW 596.67), EDC (12.84 mg, 0.067 mmol) and NHS (7.71 mg, 0.067 mmol) were added to deionized water (10 ml) and stirred for 30 min until completely dissolved. Then, the purified GSH-CDs (40 mg) were added to the mixed solution and stirred for 24 h. The final solution was lyophilized to obtain GSH-CDs-Ce6. The entire synthesis process was shielded from light.

### Enzyme-like activity of GSH-CDs

The superoxide dismutase (SOD)-like activity of the GSH-CDs in removing superoxide anions (·O_2_^−^) was evaluated according to the instructions of the total superoxide dismutase assay kit with nitrogen blue tetrazole (NBT). The detection principle is summarized as follows. The reaction system between xanthine and xanthine oxidase produces ·O_2_^−^, which can reduce NBT to blue formazan, and the latter shows strong absorption at 560 nm. Therefore, the activity level of superoxide dismutase can be calculated by colorimetry.

H_2_O_2_ scavenging assay: The catalase (CAT)-like activity of the GSH-CDs (100 µg mL^− 1^) was evaluated with H_2_O_2_ (0.8 M) in neutral sodium acetate buffer solution. After mixing, the released O_2_ was measured using a dissolved oxygen meter (Mettler Toledo, Switzerland).

The glutathione peroxidase (GPx)-like activity of the GSH-CDs was evaluated by a total glutathione peroxidase assay kit. This kit utilizes an indirect method of measurement. GPx catalyzed the production of GSSG from GSH, while glutathione reductase catalyzed the production of GSH from GSSG utilizing NADPH, and the level of glutathione peroxidase activity could be calculated by measuring the reduction in NADPH.

The total antioxidant capacity of the GSH-CDs was estimated by a total antioxidant capacity detection kit with a rapid ABTS method. ABTS was oxidized to green ABTS^+^ under the action of appropriate oxidants, and the production of ABTS^+^ could be inhibited in the presence of antioxidants. Hence, the total antioxidant capacity of the GSH-CDs was determined by measuring the absorbance of ABTS^+^ at 734 nm–405 nm.

### Extraction and cultivation of primary rat NPCs

Ten SD rats (8 weeks old, male) were sacrificed with an overdose of 2% (w/v) pentobarbital sodium. NP tissue from the caudal vertebra of rats was collected and digested with 0.5% collagenase type II at 37 °C overnight. Afterward, the suspension was centrifuged for 3 min at 1200 rpm, and the sediment was incubated with complete DMEM/F12 containing 10% fetal bovine serum and 1% penicillin/streptomycin. Then, the NPCs were cultured in an incubator with 5% CO_2_ at 37 °C. The medium was renewed every other day, and the first three generations of NPCs were used in the experiments. The newly extracted NPCs were identified via toluidine blue staining and aggrecan immunofluorescence staining.

### Cell viability test and calcein/PI staining

**A** CCK-8 kit was used to detect cell viability in vitro. NPCs were seeded into 96-well plates at a density of 8,000 cells/well and cultured at 37 °C for 24 h under 5% CO_2_. Then, the cells were treated with different concentrations of GSH-CDs or H_2_O_2_ for 24 h, and cell viability was tested utilizing a CCK-8 kit. To explore the ability of GSH-CDs to rescue H_2_O_2_-induced oxidative stress injury in NPCs, NPCs were seeded into 96-well plates at a density of 8000 cells/well, and treated with different concentrations of GSH-CDs for 30 min. Then, the cells were cocultured with H_2_O_2_ (100 µM) for 24 h. Finally, cell viability was determined by CCK-8 assay. Furthermore, following the same treatment protocol, the experiment was validated in 12-well plates (2 × 10^4^ cells/well) with calcein/PI staining solution, and the ability of the GSH-CDs to rescue the NPCs was confirmed via fluorescence microscopy (Carl Zeiss, Germany).

### Assessment of intracellular ROS levels

The DHE red fluorescent probe was used to detect intracellular ROS levels. Rat NPCs (2 × 10^4^ cells/well in a 12-well plate) were first incubated with GSH-CDs for 30 min and then cocultured with H_2_O_2_ (100 µM) or PBS for 24 h. Then, the cells were rinsed three times with PBS before the DHE fluorescence probe was loaded for 20 min at 37 °C. Finally, after nuclear staining with DAPI for 5 min, the NPCs were washed three times with PBS and imaged with a fluorescence microscope.

### Mitochondrial membrane potential staining

Mito-Tracker Red CMXRos staining was used to detect the intracellular mitochondrial membrane potential (MMP), which can effectively reflect the functional state of mitochondria. NPCs incubated with different concentrations of GSH-CDs for 30 min were then cocultured with H_2_O_2_ (100 µM) or PBS for 24 h. Next, the cells were gently washed three times with PBS and incubated with CMXRos (100 nM) at 37 °C for 20 min. Finally, NPCs were stained with DAPI for 5 min after fixation and permeabilization and observed under a fluorescence microscope.

### Cellular senescence-related staining

A senescence-associated β-galactosidase (SA-β-Gal) staining kit was utilized to determine the senescence level of NPCs. After treatment with different concentrations of GSH-CDs and H_2_O_2_ for 24 h, the NPCs were gently washed three times with PBS. Then, after fixation, 1 mL of SA-β-Gal staining solution prepared according to the instructions was added to each well overnight at 37 °C. Finally, NPCs were observed under an optical microscope.

### Immunofluorescence staining

Interventions in different groups were repeated as described above after NPCs were seeded in a 24-well plate (3 × 10^4^ cells/well). After 4% paraformaldehyde fixation, 0.3% Triton X-100 permeabilization, and gentle rinsing with PBS, the NPCs were blocked for 2 h at room temperature. Then, 200 µL of the following diluted primary antibodies (anti-TNF-α, anti-p21, anti-Aggrecan, and anti-MMP13) were added to each well and incubated overnight at 4°C. The next day, the NPCs were treated with the corresponding fluorescent secondary antibody (goat anti-mouse IgG H&L, goat anti-rabbit IgG H&L) at 37 °C for 2 h after being gently washed three times with PBS. Finally, after DAPI staining of the nucleus, photographs were obtained using a fluorescence microscope.

### Animal and surgical procedures

The Yangzhou University School of Medicine’s Ethics Committee’s norms and regulations were followed in all animal operations (202,402,101). Thirty-two male Sprague‒Dawley (SD) rats (4 weeks old) were randomly divided into four groups to evaluate the rescue effect of local injection of GSH-CDs in vivo: the normal control group (NC group), degeneration control group (Vehicle group), L-GSH-CD group (7.14 × 10^− 4^ mg kg^− 1^), and H-GSH-CD group (1.43 × 10^− 3^ mg kg^− 1^). After intraperitoneal injection of pentobarbital sodium for anesthetization and tail surface disinfection, the disc was punctured with a 21G needle between the 8th and 9th caudal vertebra (Co8–9). For the treatment, 10 µL of the following solutions were immediately injected into the rat IVD after needling via a microsyringe: the NC group (no intervention), vehicle group (PBS), L-GSH-CD group (7.14 × 10^− 4^ mg kg^− 1^), and H-GSH-CD group (1.43 × 10^− 3^ mg kg^− 1^). After experiencing regular changes in feed, bedding and water, all rats were narcotized for X-ray and MRI examination four weeks after the operation and then sacrificed for tissue sectioning of the Co8-9 intervertebral disc.

### X-ray and MRI analysis

The rats were kept in the prone position with their tails extended and placed on X-ray imaging equipment (GE SIGNA Architect). Coronal T_2_-weighted MR images were acquired to evaluate signal and structural differences in the intervertebral disc. The IVD heights and adjacent upper and lower vertebral body heights were obtained from radiographic parameters to calculate the disc height index (DHI). The water content of the rat NP tissue was reflected via the mean gray values (MGVs) on the coronal T_2_-weighted images.

### In vivo biocompatibility and fluorescence metabolism

The same dose (5 mg kg^− 1^) of PBS or GSH-CDs was injected into the tail vein of male BALB/c mice (purchased from Yangzhou University’s Laboratory Animal Centre). The mice were euthanized after receiving therapy for thirty days, and fresh blood was drawn and subjected to biochemical and hematological tests. HE staining was performed on the main organs of the mice, such as the heart, liver, spleen, lung and kidney, for biosafety evaluation. In addition, GSH-CDs-Ce6 (5 mg kg^− 1^) were injected into the tail vein of mice, and in vivo animal images were captured at different time points after injection to verify the distribution and metabolic trends of the GSH-CDs-Ce6 in vivo.

### Tissue morphology assessment

After fixation, complete decalcification and dehydration, the collected rat tail vertebral specimens were embedded in paraffin and then cut into 8 μm sections from the coronal plane. For histological assessment, paraffin tissue sections were stained with hematoxylin and eosin (HE) and Safranin O-Fast Green (SOFG) according to standard laboratory protocols. In addition, with reference to Masuda’s previous study to calculate histological grades, the histological grading system (Table 1) was divided into four categories, ranging from 4 (normal) to 12 points (severe degeneration).

**Table 1:** Histological grading system of intervertebral disc

Category	Grade
Annulus fibrosus	1. Annularly arranged fiber ring, without terminations or twists.
	2. Interruption or distortion below 30%.
	3. Interruption or distortion over 30%.
Demarcation of the annulus	1. Clear demarcation fibrosus from nucleus pulposus.
	2. Slight interruption in demarcation.
	3. Heavy integration of demarcation.
Number and morphology of NPCs	1. Normal morphology, rich in NPCs, abundant extracellular matrix.
	2. Slight decrease in the number of NPCs.
	3. Significant cell loss (more than 50%).
Extracellular matrix of NPCs	1. Extracellular matrix in normal gel-like form.
	2. Slight coagulation of extracellular matrix.
	3. Severe coagulation of extracellular matrix.

### Statistical analysis

All experimental data are presented as the mean ± standard deviation (SD). The original data were analyzed by using GraphPad Prism 9.00 (GraphPad Software, USA). Student’s t test (t test) was used for comparisons between two groups, and one-way analysis of variance (ANOVA) was used for comparisons among more than two groups. A P value less than 0.05 was considered to indicate statistical significance (* indicates *p* < 0.05, ** indicates *p* < 0.01, *** indicates *p* < 0.001, **** indicates *p* < 0.0001, ns indicates not significant).

## Conclusion

In summary, the novel carbon dot nanozyme GSH-CDs were characterized by a simple preparation process, with high enzyme-like activity and good biocompatibility. In brief, GSH-CDs possess excellent antioxidant-like enzyme activities such as SOD, CAT, and GPx, which can effectively remove ROS. Thus, GSH-CDs are able to effectively reduce the senescence level of NPCs via scavenging excess ROS and exerting antioxidative stress effects in vitro, thus reversing the progression of IVDD in vivo. During this treatment, GSH-CDs also effectively inhibited the expression of SASP in NPCs and promoted the anabolic process. In addition, GSH-CDs exhibit excellent biocompatibility and can be completely metabolized within 12 h after intravenous injection. Consequently, it can be tentatively considered to show greater research value and clinical translation potential, providing a new theoretical basis for IVDD management. In addition, due to the prominent antioxidant effect of GSH-CDs, they may possess considerable value for application in other diseases.

## Data Availability

No datasets were generated or analysed during the current study.
